# Supplementary material for: Synergistic cancer immunotherapy combines MVA-CD40L induced innate and adaptive immunity with tumor targeting antibodies
Source: Nat Commun. 2019 Nov 6;10:5041. doi: 10.1038/s41467-019-12998-6 (PMC6834557; doi:10.1038/s41467-019-12998-6)
Supplement: Supplementary file 1 — Supplementary Information [file 41467_2019_12998_MOESM1_ESM.pdf]

Synergistic cancer immunotherapy combines MVA CD40L induced innate and adaptive immunity with tumor targeting antibodies

´Medina-Echeverz et al.´

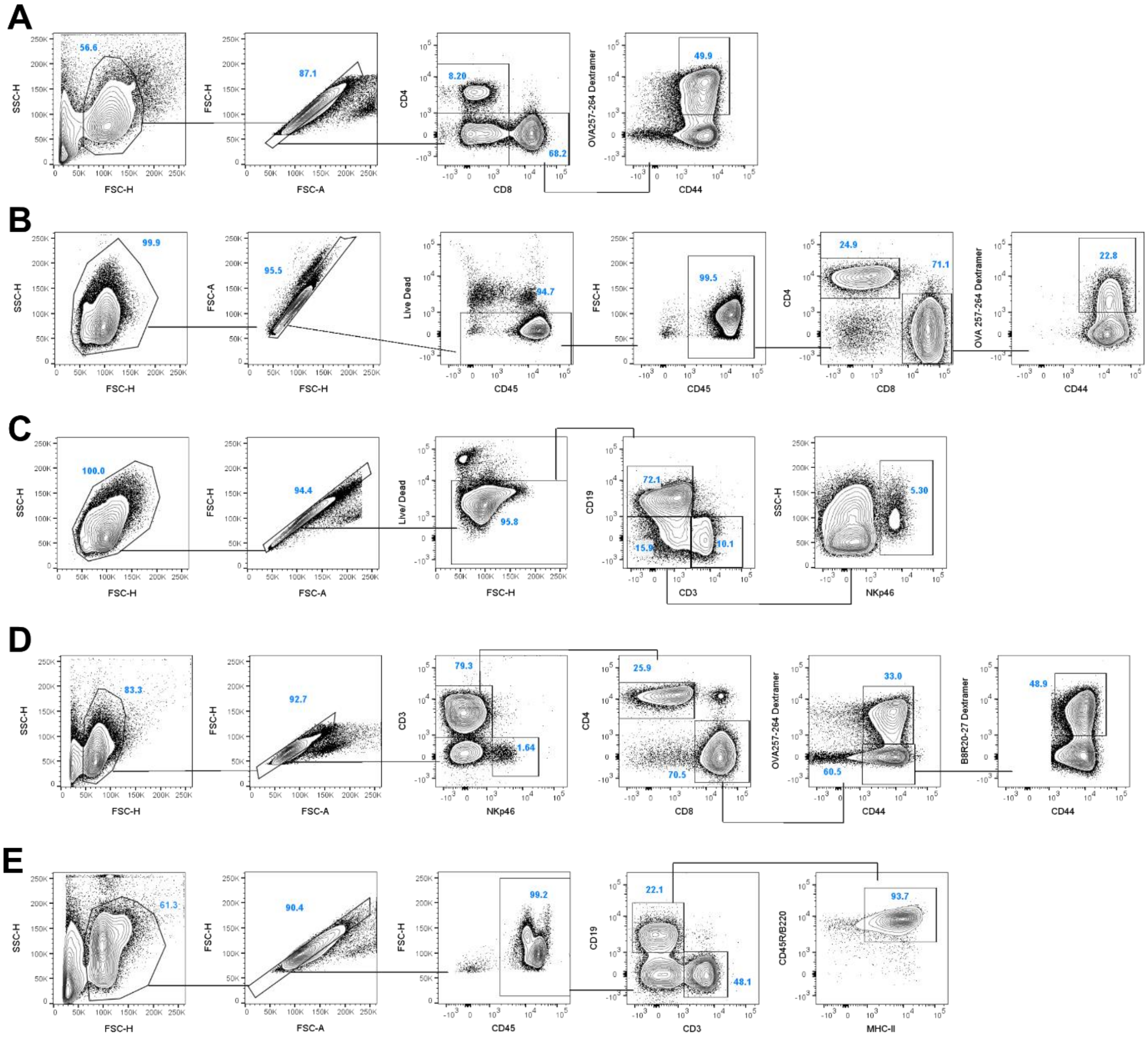

Supplementary Figure 1. Gating strategies for flow cytometry analysis. (A) Related to *Supplementary Figure 2*. Gating strategy on blood peripheral leukocytes to check percentage of OVA$_{257-264}$ Dextramer$^+$ CD8$^+$ T cells upon rMVA-CD40L. immunization; **(B)** Related to *Figure 2.* Gating strategy on tumor cell suspensions seven days upon rMVA-CD40L. immunization to check percentage of CD4$^+$ and OVA$_{257-264}$ Dextramer$^+$ CD8$^+$ T cell proliferation and exhaustion phenotype; **(C)** Related to *Figure 4* and *Supplementary Figure 5.* Representative gating strategy showed in splenic single cell suspensions to analyze NK cell frequencies and phenotype; **(D)** Related to *Supplementary Figure 7*. Gating strategy on blood peripheral leukocytes to check percentage of NK cells, OVA$_{257-264}$ Dextramer$^+$ CD8$^+$ T cells and B8R$_{20-27}$ Dextramer$^+$ CD8$^+$ T cells upon rMVA-CD40L. immunization; **(E)** Related to *Supplementary Figure 8*. Gating strategy on blood peripheral leukocytes to check percentage of B cells upon rMVA-CD40L immunization.

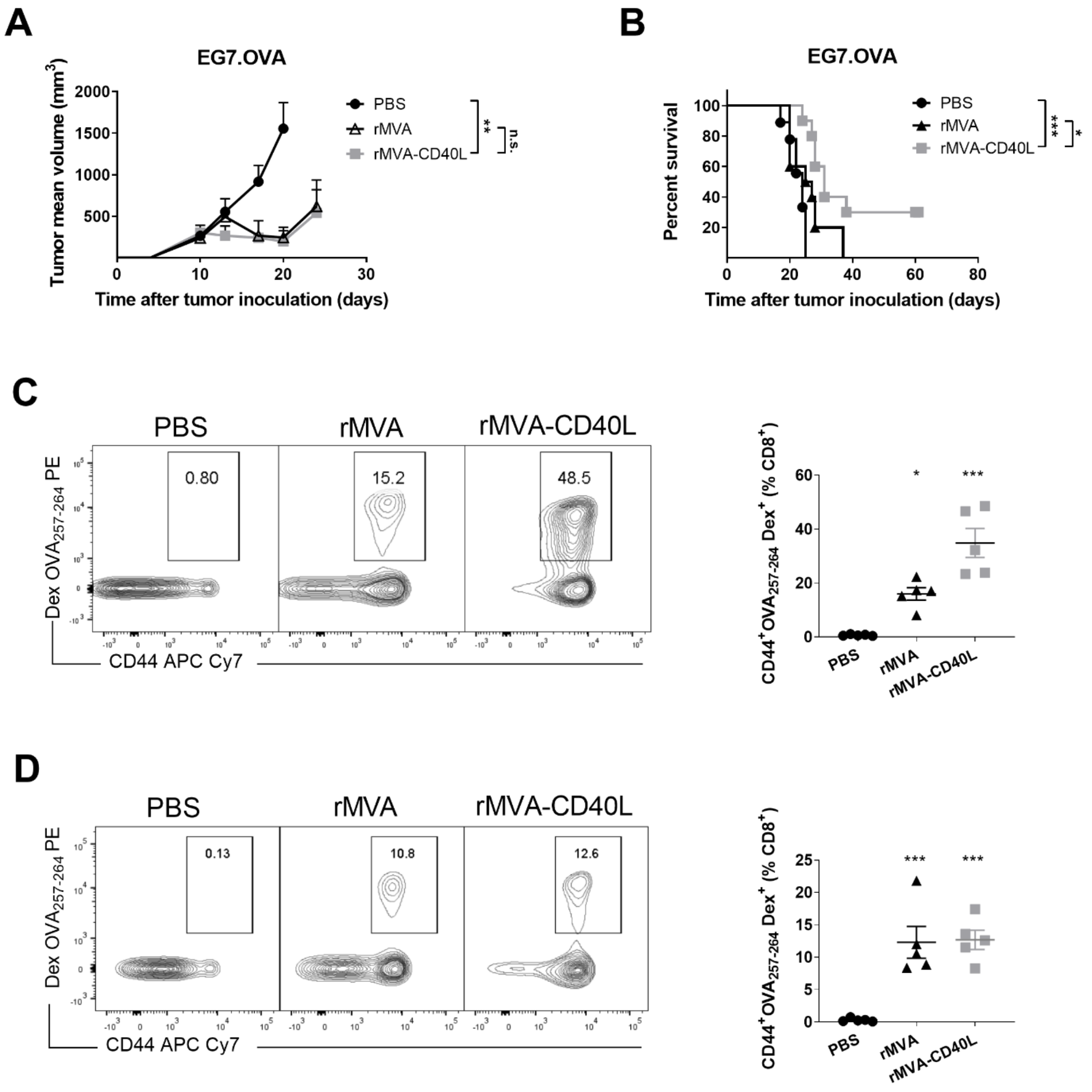

Supplementary Figure 2. Therapeutic efficacy in EG7.OVA lymphoma and increased peripheral blood CD8$^+$ T cell responses in rMVA-CD40L immunized tumor-bearing mice. Related to *Figure 1*. C57BL/6 mice received EG7.OVA as described at *Figure 1A*. **(A)** Tumor size follow-up (n=5 mice/group) and **(B)** overall survival (n= 10 mice/group) of mice injected either with PBS, MVA-OVA (referred to as rMVA) or MVA-OVA-CD40L (referred to as rMVA-CD40L); **(C)** Representative dot plots and frequency of peripheral blood CD44$^+$ Tet OVA$_{257\text{-}264}^+$ CD8$^+$ T cells 7 days after PBS, rMVA or rMVA-CD40L immunization to B16.OVA tumor-bearing mice (n= 5 mice/group). Data are representative of at least 2 independent experiments. **(D)** Representative dot plots and frequency of peripheral blood CD44$^+$ Dex OVA$_{257\text{-}264}^+$ CD8$^+$ T cells 7 days after PBS, rMVA or rMVA-CD40L immunization of EG7.OVA tumor-bearing mice (n= 5 mice/group). Data are representative of at least two independent experiments. Data expressed as Mean ± SEM. One-way ANOVA comparing MVA immunization vs PBS. *$p < 0.05$, ***$p < 0.005$.

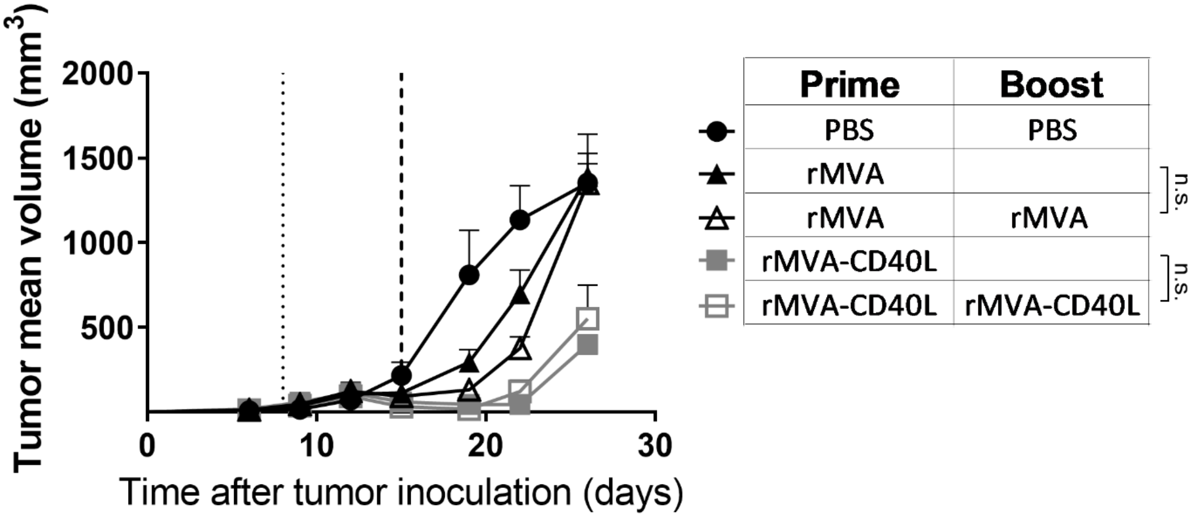

Supplementary Figure 3. Repeated intravenous administration of rMVA vectors did not increase therapeutic efficacy. Briefly, C57BL/6 mice received B16.OVA cells subcutaneously in the flank. Seven days later, when tumors were above 50 mm^3, mice were grouped (n=5 mice/group) and immunized intravenously either with PBS or with $5x10^7$ $TCID_{50}$ of the mentioned rMVA viruses in the table (black dotted line). Seven days after prime immunization, tumor-bearing mice received a boost immunization with $5x10^7$ $TCID_{50}$ of the mentioned rMVA viruses in the table when indicated (black dashed line). Tumor growth was measured at regular intervals. Data expressed as Mean ± SEM. One-way ANOVA comparing MVA immunization vs PBS. *$p < 0.05$, ***$p < 0.005$.

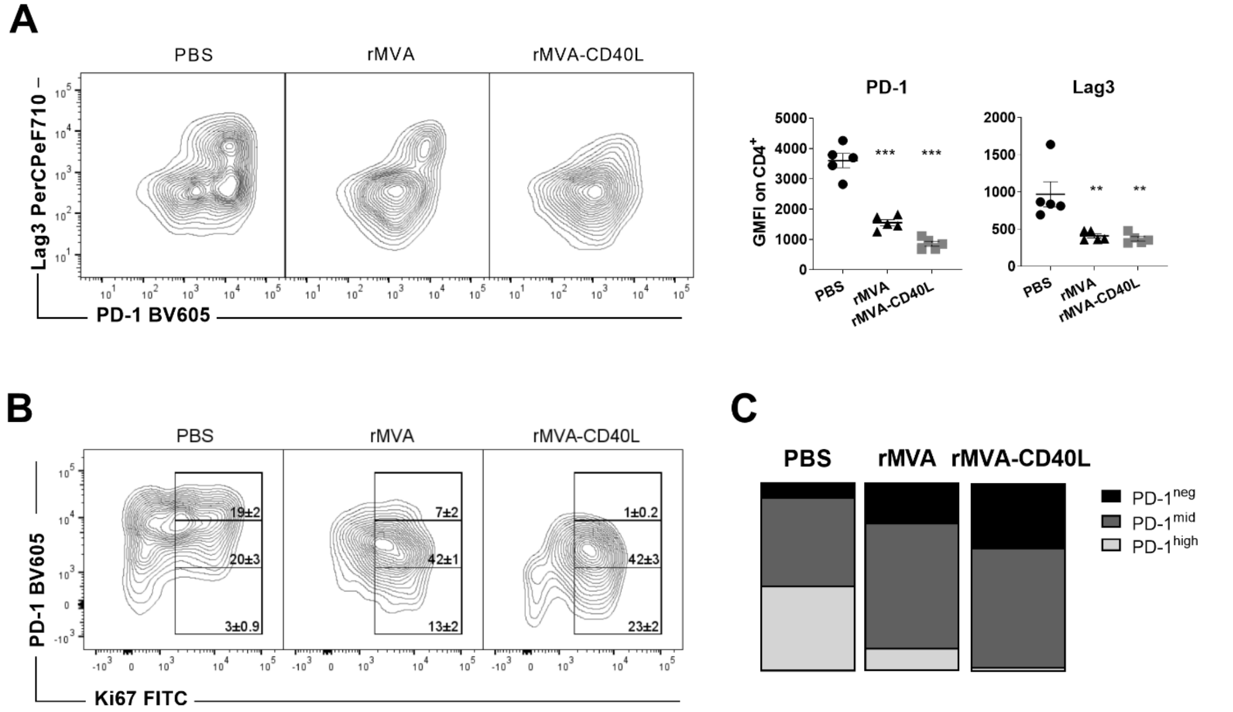

Supplementary Figure 4. Tumor-infiltrating CD4 T cells present a less exhausted and proliferating phenotype upon treatment. B16.OVA tumor bearers were immunized when tumors reached at least 50 mm^3 in volume. Seven days later, mice were sacrificed for further analysis (n= 5 mice/group). **(A)** Representative dot plots of PD-1 and Lag3 co-expression in tumor-infiltrating $CD4^+$ T cells; **(B)** GMFI of PD-1 and Lag3 on tumor-infiltrating $CD4^+$ T cells showing Mean ± SEM, representative of at least two independent experiments; **(C)** Representative dot plot of Ki67 and PD-1 expression on tumor-infiltrating $CD4^+$ T cells showing Mean ± SEM, representative of at least two independent experiments. B, One-way ANOVA comparing treatment groups vs PBS. *, *p* <0.05; **, *p* <0.01; ***, *p* <0.005.

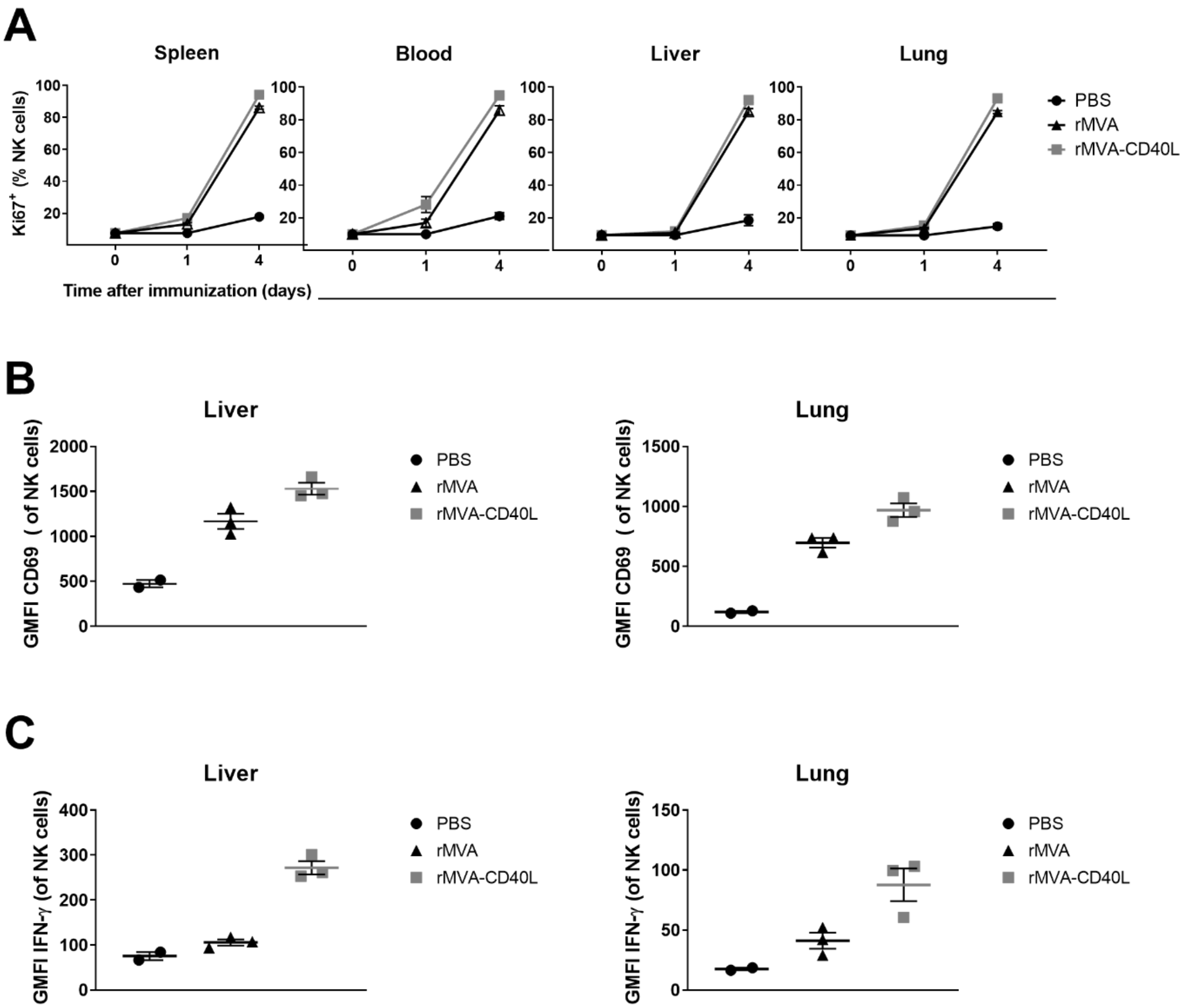

Supplementary Figure 5. Intravenous immunization with rMVA CD40L enhances NK cell proliferation and activation in liver and lung. (A) Systemic proliferation of NK cells upon intravenous rMVA immunization. C57BL/6 mice received PBS (n=2 mice) or were immunized i.v. with $5x10^7$ $TCID_{50}$ of either rMVA or rMVA-CD40L (n= 3 mice/group). Mice were sacrificed at days 1 and 4 after immunization and the frequency of Ki67-positive NK cells (defined as $CD3^-$ $NKp46^+$ cells) in spleen, blood, liver and lung was assessed. Data are expressed as Mean ± SEM, representative of two independent experiments; **(B, C)** rMVA or rMVA-CD40L induction of NK cell activation. C57BL/6 mice received PBS (n=2 mice) or were immunized i.v. with $5x10^7$ $TCID_{50}$ of either rMVA or rMVA-CD40L (n= 3 mice/group). Mice were sacrificed at day 1 after immunization and the expression of CD69 **(B)** and IFN-γ **(C)** on liver and lung-infiltrating NK cells was analyzed by flow cytometry. Data are expressed as Mean ± SEM, representative of two independent experiments.

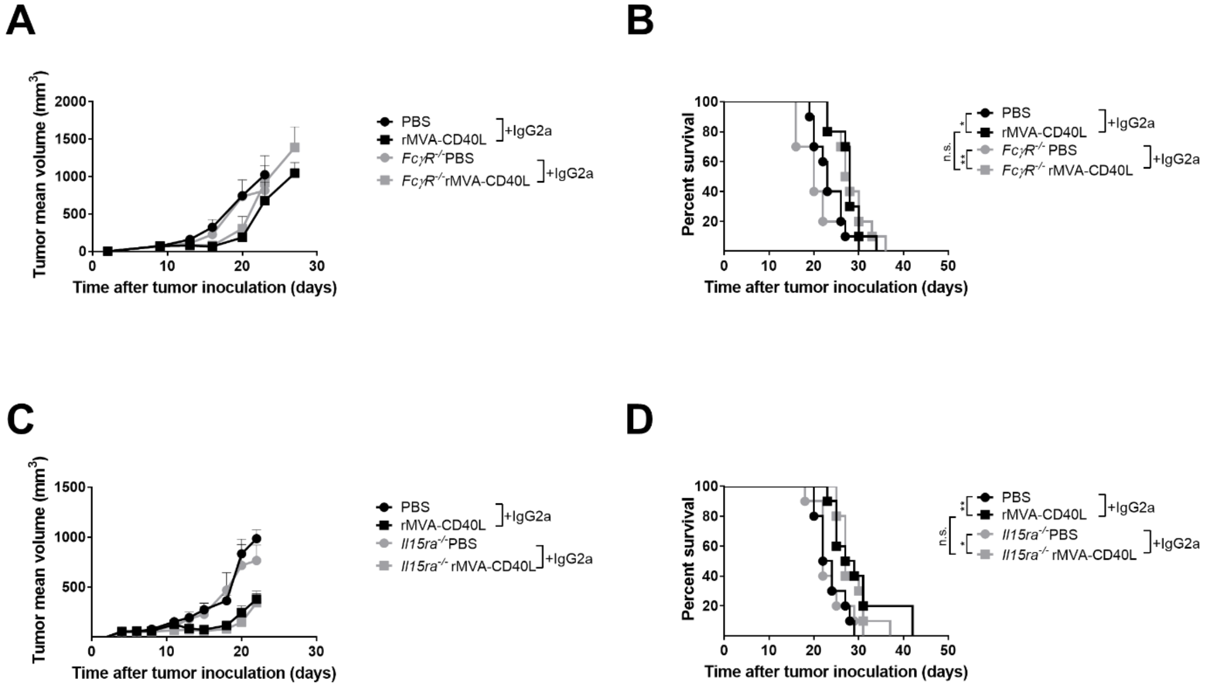

Supplementary Figure 6. rMVA-CD40L enhances tumor growth control both in *FcγR*$^{-/-}$ and *IL15ra*$^{-/-}$ tumor-bearing mice. (A, B) Performed along with Figures 6A and 6B. B16.OVA tumor-bearing wild type and *FcγR*$^{-/-}$ mice were grouped according to tumor size. Tumor-bearing mice either received PBS or were immunized with $5x10^7$ $TCID_{50}$ of rMVA-CD40L (Day 0). Mice received 200 µg of IgG2a antibody i.p. at days -2, 2, 6 and 10; **(A)** Tumor size follow-up (n= 5 mice/group) and **(B)** overall survival (n=10 mice/group). **(C, D)** Performed along with Figures 6C and 6D. B16.OVA tumor-bearing wild type and *Il15ra*$^{-/-}$ mice were grouped according to tumor size. Tumor bearing littermates either received PBS or were immunized with $5x10^7$ $TCID_{50}$ of rMVA-CD40L (Day 0). Mice received 200 µg of IgG2a antibody i.p. at days -2, 2, 6 and 10; **(C)** Tumor size follow-up (n= 5 mice/group) and **(D)** overall survival (n=10 mice/group). B and D represent overall survival of two merged independent experiments. Data in A and C are expressed as Mean ± SEM. One-way ANOVA was performed on figures A and C. Log rank test on mouse survival was performed for figures B and D. *, $p < 0.05$; **, $p < 0.01$; n.s. non-significant.

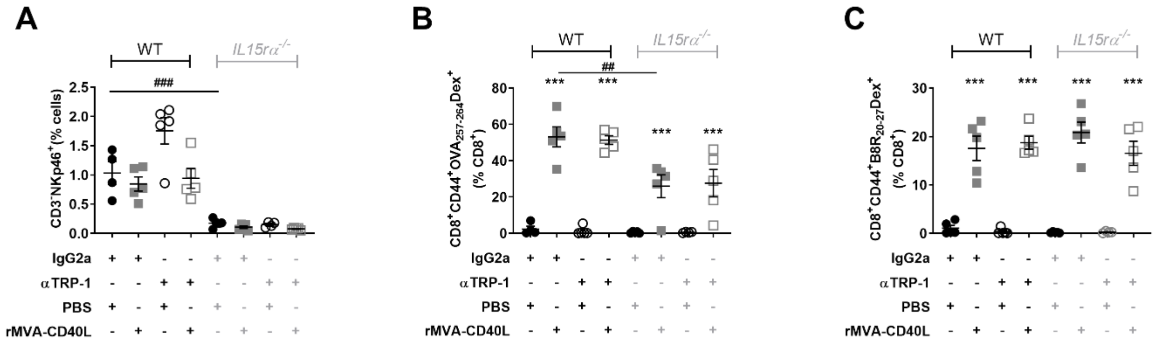

Supplementary Figure 7. rMVA-CD40L expands antigen-specific CD8$^+$T cells in the absence of IL15Rα. WT and *IL15rα*$^{-/-}$ B16.OVA tumor bearing mice (n=5 mice/group) were treated as described in Figures 5 and 6. Seven days after immunization, mice were bled and peripheral blood analysis of immune cell subsets by flow cytometry was conducted. **(A)** Percentage of NK cells-gated as CD3$^-$NKp46$^+$- in peripheral blood; **(B)** Frequency of OVA-specific CD8 T cells in peripheral blood; **(C)** Frequency of MVA-specific CD8 T cells in peripheral blood). Data in A, B and C are representative of two independent experiments. Data expressed as Mean ± SEM. One-way ANOVA. * represents the statistically significant differences among treatment groups from the same genotype, whereas # represents statistically significant differences among the same treatment group compared between wild type and *Il15Rα*$^{-/-}$ littermates. $^{\#\#}p < 0.01$, $^{\#\#\#}p < 0.05$, $^{***}p < 0.005$.

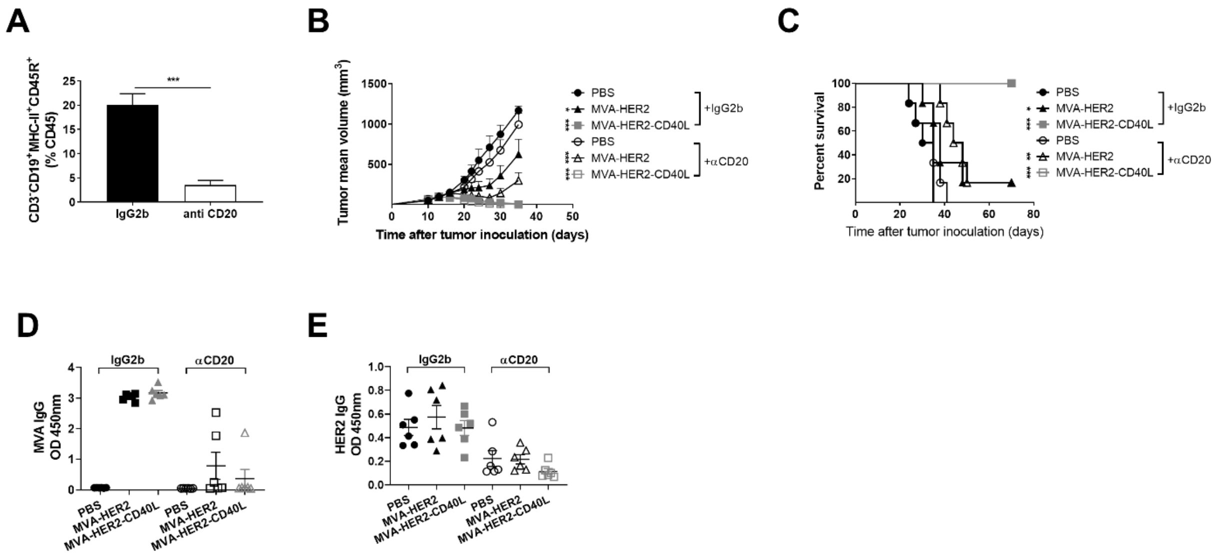

Supplementary Figure 8. B cells and B cell-mediated antibodies are dispensable for rMVA-CD40L antitumor effect. Balb/c mice received 5x10^5 CT26.HER2 cells subcutaneously. 13 days later, mice were grouped (n=6 mice/group) and IV injected either with PBS, 5×10^7 TCID$_{50}$ MVA-HER2 or 5×10^7 TCID$_{50}$ MVA-HER2-CD40L. Mice received 250µg anti-CD20 Ab (clone SA271G2) or IgG2b IV 4 days prior and 9 days after tumor cell inoculation. Blood for the detection of specific antibodies was withdrawn 7 days before and 27 days after CT26.HER2 tumor cell inoculation. Tumor growth was measured at regular intervals. **(A)** Percentage of CD3$^-$ CD19$^+$ MHC-II$^+$ CD45R$^+$ cells of CD45$^+$ cells in blood 8 days after CT26.HER2 cell inoculation; **(B)** Mean tumor volume; **(C)** Kaplan Meier plot showing survival of mice. ELISA on serum samples taken at day 27 after tumor inoculation against MVA IgG **(D)** or HER2 IgG **(E)**. This experiment has been partially shown in Figures 1H and 1I. Data expressed as Mean ± SEM. B was analyzed using One-way ANOVA comparing MVA immunization vs PBS; C was analyzed using Log-rank test. *$p < 0.05$, ***$p < 0.005$.
