# Supplementary material for: Synergistic cancer immunotherapy combines MVA-CD40L induced innate and adaptive immunity with tumor targeting antibodies
Source: Nat Commun. 2019 Nov 6;10:5041. doi: 10.1038/s41467-019-12998-6 (PMC6834557; doi:10.1038/s41467-019-12998-6)
Supplement: Supplementary file 3 — Reporting Summary [file 41467_2019_12998_MOESM3_ESM.pdf]

nature research

Corresponding author(s): Hubertus Hochrein

Last updated by author(s): 04-October-2019

Reporting Summary

Nature Research wishes to improve the reproducibility of the work that we publish. This form provides structure for consistency and transparency in reporting. For further information on Nature Research policies, see Authors & Referees and the Editorial Policy Checklist .

Statistics

For all statistical analyses, confirm that the following items are present in the figure legend, table legend, main text, or Methods section.

n/a	Confirmed	
[ ]	[x]	The exact sample size (*n*) for each experimental group/condition, given as a discrete number and unit of measurement
[ ]	[x]	A statement on whether measurements were taken from distinct samples or whether the same sample was measured repeatedly
[ ]	[x]	The statistical test(s) used AND whether they are one- or two-sided<br>*Only common tests should be described solely by name; describe more complex techniques in the Methods section.*
[x]	[ ]	A description of all covariates tested
[ ]	[x]	A description of any assumptions or corrections, such as tests of normality and adjustment for multiple comparisons
[ ]	[x]	A full description of the statistical parameters including central tendency (e.g. means) or other basic estimates (e.g. regression coefficient) AND variation (e.g. standard deviation) or associated estimates of uncertainty (e.g. confidence intervals)
[x]	[ ]	For null hypothesis testing, the test statistic (e.g. *F*, *t*, *r*) with confidence intervals, effect sizes, degrees of freedom and *P* value noted<br>*Give P values as exact values whenever suitable.*
[x]	[ ]	For Bayesian analysis, information on the choice of priors and Markov chain Monte Carlo settings
[x]	[ ]	For hierarchical and complex designs, identification of the appropriate level for tests and full reporting of outcomes
[x]	[ ]	Estimates of effect sizes (e.g. Cohen's *d*, Pearson's *r*), indicating how they were calculated

Our web collection on statistics for biologists contains articles on many of the points above.

Software and code

Policy information about availability of computer code

Data collection	BD FACS Diva for flow cytometry: BD FACS DIVA software, Version 8.0.2 and BD FACS DIVA software, Version 6.1.1, BD Biosciences<br>Luminex MAGPIX: xPONENT, Build 4.2.1324.0, Luminex Corporation
Data analysis	Provided in the manuscript; commercial software such as FlowJo software (Tree Star) FlowJo 10.3;<br>Masterplex 2010 version 2.0.0.77, Module MasterPlex QT (Ver. 5.0.0.77) Hitachi Solutions, Ltd.

For manuscripts utilizing custom algorithms or software that are central to the research but not yet described in published literature, software must be made available to editors/reviewers. We strongly encourage code deposition in a community repository (e.g. GitHub). See the Nature Research guidelines for submitting code & software for further information.

Data

Policy information about availability of data

All manuscripts must include a data availability statement. This statement should provide the following information, where applicable:
- Accession codes, unique identifiers, or web links for publicly available datasets
- A list of figures that have associated raw data
- A description of any restrictions on data availability

The authors declare that the data supporting the findings of this study are available within the paper and its supplementary information files or available from the authors upon reasonable request.

Field-specific reporting

Please select the one below that is the best fit for your research. If you are not sure, read the appropriate sections before making your selection.

Life sciences study design

All studies must disclose on these points even when the disclosure is negative.

Sample size

Sample size used for in vivo experiments is n=5 mice per experimental group, determined by experience with the tumor models used and confirmed useful for the limited availability of knockout mice. When naive mice were used, sample sizes varies between PBS (n=2 mice) and treated mice (n=3 to 5 mice when indicated).

Data exclusions

No data were excluded from the analysis.

Replication

In vivo results were repeated at least twice for reproducibility. All attempts of replication were successful. When results were not repeated, it is clearly stated in the manuscript.

Randomization

For in vivo experiments, mice were randomized by the investigators according to the measured tumor mean diameter and ultimately by the tumor mean volume. So, the intrinsic variability of tumor cell growth in vivo is equally spread among the groups.

Blinding

Experiments were not blinded. Technical research personnel preparing the treatment groups and performing the experiments were aware of which group receives a particular treatment. In addition, the local authorities require that, for in vivo experiments, the substances used are written onto the cage cards. Finally, all samples processed and assayed by the technical research personnel are handled the same way.

Reporting for specific materials, systems and methods

We require information from authors about some types of materials, experimental systems and methods used in many studies. Here, indicate whether each material, system or method listed is relevant to your study. If you are not sure if a list item applies to your research, read the appropriate section before selecting a response.

Materials & experimental systems

n/a	Involved in the study
☐	☒ Antibodies
☐	☒ Eukaryotic cell lines
☐	☐ Palaeontology
☐	☒ Animals and other organisms
☐	☐ Human research participants
☐	☐ Clinical data

Methods

n/a	Involved in the study
☐	☐ ChIP-seq
☐	☒ Flow cytometry
☐	☐ MRI-based neuroimaging

Antibodies

Antibodies used

Antibody	Clone	Company	purpose	Cat.No.	Fluorophore
CD3	17A2	Biolegend	Flow Cytometry	100237	BV605
CD3	145-2C11	Biolegend	Flow Cytometry	100335	BV421
CD4	RM4-5	Biolegend	Flow Cytometry	100545	BV650
CD8α	53-6.7	Biolegend	Flow Cytometry	100749	BV785
CD19	1D3	Thermo Fisher	Flow Cytometry	25-0193-82	PE/Cy7
CD44	IM7	Thermo Fisher	Flow Cytometry	47-0441-82	APC-eFluor780
CD45	30-F11	Biolegend	Flow Cytometry	103108; 103114	FITC; PE/Cy7
CD45R/B220	RA3-6B2	Thermo Fisher	Flow Cytometry	45-0452-82	PerCP Cy5.5
CD69	H1.2F3	Biolegend	Flow Cytometry	104527	BV421
CD70	FR70	Thermo Fisher	Flow Cytometry	46-0701-82	PerCP-eFluor710
CD223 (Lag3)	C9B7W	Thermo Fisher	Flow Cytometry	46-2231-82	PerCP-eFluor710
CD279 (PD-1)	29F.1A12	Biolegend	Flow Cytometry	135219	BV605
CD279 (PD-1)	RMP1-30	Thermo Fisher	Flow Cytometry	17-9981-82	APC
CD335 (NKp46)	29A1.4	Thermo Fisher	Flow Cytometry	46-3351-82	PerCP-eFluor710
Ki67	35/Ki-67	BD	Flow Cytometry	556026	FITC
IFN-g	XMG1.2	Thermo Fisher	Flow Cytometry	25-7311-41	PE/Cy7
FoxP3	FJK-16s	Thermo Fisher	Flow Cytometry	17-5773-82	APC
MHC-II (Ia/Ie)	M5/114.15.2	Thermo Fisher	Flow Cytometry	47-5321-82	PerCP eFluor780
OVA-SIINFEKL	Custom	Immudex	Flow Cytometry		PE
B8R-TSYKFESV	Custom	Immudex	Flow Cytometry		APC
Trastuzumab	Tra-huIgG1	InvivoGen	In vivo treatment	her2tra-mab1	
β-Gal	Bgal-huIgG1	InvivoGen	In vivo treatment	bgal-mab1	
anti TRP-1	TA99	B-XCell	In vivo treatment	BE0151	
mouse IgG2a	2A3	B-XCell	In vivo treatment	BE0085	

mouse IgG2b	LTF2	B-XCell	In vivo treatment	BE0090
Anti CD8	2.47	B-XCell	In vivo treatment	BE0061
Anti CD20	SA271G2	Biolegend	In vivo treatment	152104

Validation

Antibody Clone Company Aplication species/reactivity Test in manufacturer´s description
CD45 30-F11 Biolegend Flow Cytometry Rat anti-mouse staining of Balb/c splenocytes
CD3 17A2 Biolegend Flow Cytometry Rat anti-mouse staining of C57BL/6 splenocytes
CD3 145-2C11 Biolegend Flow Cytometry Rat anti-mouse staining of C57BL/6 splenocytes
CD4 RM4-4 Biolegend Flow Cytometry Rat anti-mouse staining of C57BL/6 splenocytes
CD8α 53-6.7 Biolegend Flow Cytometry Rat anti-mouse staining of C57BL/6 splenocytes
CD19 1D3 Thermo Fisher Flow Cytometry Rat anti-mouse staining of Balb/c splenocytes
CD44 IM7 Thermo Fisher Flow Cytometry Rat anti-mouse staining of C57BL/6 splenocytes
CD45 30-F11 Biolegend Flow Cytometry Rat anti-mouse staining of C57BL/6 splenocytes
CD45R/B220 RA3-6B2 Thermo Fisher Flow Cytometry Rat anti-mouse staining of C57BL/6 splenocytes
CD69 H1.2F3 Biolegend Flow Cytometry Rat anti-mouse PMA/iono stimulation of C57BL/6 splenocytes (6 hrs)
CD70 FR70 Thermo Fisher Flow Cytometry Rat anti-mouse LPS and anti CD40 stimulation of mouse splenocytes
CD223 (Lag3) C9B7W Thermo Fisher Flow Cytometry Rat anti-mouse 3-day anti-mouse CD3 and anti-mouse CD28 stimulated splenocytes
CD279 (PD-1) 29F.1A12/RMP1-30 Biolegend Flow Cytometry Rat anti-mouse ConA-stimulated C57BL/6 splenocytes (three days)
CD335 (NKp46) 29A1.4 Thermo Fisher Flow Cytometry Rat anti-mouse staining of C57BL/6 splenocytes
Ki67 35/Ki-67 BD Flow Cytometry Reactivity human/mouse intracellular staining as test
IFN-g XMG1.2 Thermo Fisher Flow Cytometry Rat anti-mouse Intracellular staining of stimulated mouse splenocytes
FoxP3 FJK-16s Thermo Fisher Flow Cytometry Rat anti-mouse/human intracellular staining of splenocytes
MHC-II (Ia/Ie) M5/114.15.2 Thermo Fisher Flow Cytometry Rat anti-mouse staining of balb/c and C57BL/6 splenocytes
OVA-SIINFEKL Custom Immudex Flow Cytometry H-2Kb Dextramer Surface staining present at the paper
B8R-TSYKFESV Custom Immudex Flow Cytometry H-2Kb Dextramer Surface staining performed in our laboratory (information can be provided).
Trastuzumab Tra-huIgG1 InvivoGen In vivo treatment human Ab purified from CHO cells Binding to HER2 tested by flow cytometry in our lab (information can be provided)
β-Gal Bgal-huIgG1 InvivoGen In vivo treatment human Ab purified from CHO cells negative binding to human cells tested by manufacturer
anti TRP-1 TA99 B-XCell In vivo treatment rat anti-mouse reported in flow cytometry
mouse IgG2a 2A3 B-XCell In vivo treatment rat anti-mouse reported in flow cytometry
mouse IgG2b LTF2 B-XCell In vivo treatment rat anti-mouse reported in flow cytometry
Anti CD8 2.47 B-XCell In vivo treatment rat anti mouse in vivo antibody depletion observed in our lab (information can be provided)
Anti CD20 SA271G2 Biolegend In vivo treatment rat anti mouse in vivo antibody depletion observed in our lab (information can be provided)

Eukaryotic cell lines

Policy information about cell lines

Cell line source(s)

CT26 murine colon carcinoma cell line expressing human HER2 (CT26.HER2) was licensed from the Regents of the University of California. The B16.OVA melanoma cell line was a kind gift of Roman Spörri (University of Zürich. B16.F10 (ATCC® CRL-6475™), EG7-OVA (ATCC® CRL-2113™) and CT26.WT (ATCC® CRL-2638™) cell lines were purchased from American Type Culture Collection (ATCC). MC38.WT colon carcinoma cells were property of and used at Charles River Discovery Services.

Authentication

The cell lines used were not authenticated.

Mycoplasma contamination

All cell lines were tested negative for Mycoplasma contamination. Mycoplasma tests are ran routinely in the lab. The method of detecion used is the Lonza MycoAlert PLUS Mycoplasma Detection kit according to Standard Operating Procedures at Bavarian Nordic GmbH.

Commonly misidentified lines
(See ICLAC register)

N/A

Palaeontology

Specimen provenance

Provide provenance information for specimens and describe permits that were obtained for the work (including the name of the issuing authority, the date of issue, and any identifying information).

Specimen deposition	*Indicate where the specimens have been deposited to permit free access by other researchers.*
Dating methods	*If new dates are provided, describe how they were obtained (e.g. collection, storage, sample pretreatment and measurement), where they were obtained (i.e. lab name), the calibration program and the protocol for quality assurance OR state that no new dates are provided.*

☐ Tick this box to confirm that the raw and calibrated dates are available in the paper or in Supplementary Information.

Animals and other organisms

Policy information about studies involving animals; ARRIVE guidelines recommended for reporting animal research

Laboratory animals	6-to-8-week-old female C57BL/6J (H-2b) and Balb/cJ (H-2d) mice were purchased from Janvier Labs. Fcgr-/- and Il15ra-/- female mice were obtained from the University of Zürich. One experiment indicated in the manuscript was performed using C57BL/6J (H-2b) at Charles River Discovery Services (Morrisville, NC, USA).
Wild animals	*Provide details on animals observed in or captured in the field; report species, sex and age where possible. Describe how animals were caught and transported and what happened to captive animals after the study (if killed, explain why and describe method; if released, say where and when) OR state that the study did not involve wild animals.*
Field-collected samples	*For laboratory work with field-collected samples, describe all relevant parameters such as housing, maintenance, temperature, photoperiod and end-of-experiment protocol OR state that the study did not involve samples collected from the field.*
Ethics oversight	All animal experiments were approved by the animal ethics committee of the government of Upper Bavaria (Regierung von Oberbayern, Sachgebiet 54, Tierschutz) and were carried out in accordance with the approved guidelines for animal experiments at Bavarian Nordic GmbH. When indicated, an experiment was conducted at Charles River Discovery Services (Morrisville, NC, USA) in compliance with the Association for Assessment and Accreditation of Laboratory Animal Care International (AAALAC).

Note that full information on the approval of the study protocol must also be provided in the manuscript.

Human research participants

Policy information about studies involving human research participants

Population characteristics	*Describe the covariate-relevant population characteristics of the human research participants (e.g. age, gender, genotypic information, past and current diagnosis and treatment categories). If you filled out the behavioural & social sciences study design questions and have nothing to add here, write "See above."*
Recruitment	*Describe how participants were recruited. Outline any potential self-selection bias or other biases that may be present and how these are likely to impact results.*
Ethics oversight	*Identify the organization(s) that approved the study protocol.*

Note that full information on the approval of the study protocol must also be provided in the manuscript.

Clinical data

Policy information about clinical studies

All manuscripts should comply with the ICMJEguidelines for publication of clinical research and a completedCONSORT checklist must be included with all submissions.

Clinical trial registration	*Provide the trial registration number from ClinicalTrials.gov or an equivalent agency.*
Study protocol	*Note where the full trial protocol can be accessed OR if not available, explain why.*
Data collection	*Describe the settings and locales of data collection, noting the time periods of recruitment and data collection.*
Outcomes	*Describe how you pre-defined primary and secondary outcome measures and how you assessed these measures.*

ChIP-seq

Data deposition

☐ Confirm that both raw and final processed data have been deposited in a public database such as GEO.

☐ Confirm that you have deposited or provided access to graph files (e.g. BED files) for the called peaks.

Data access links<br>*May remain private before publication.*	*For "Initial submission" or "Revised version" documents, provide reviewer access links. For your "Final submission" document, provide a link to the deposited data.*
Files in database submission	*Provide a list of all files available in the database submission.*

Genome browser session
(e.g. UCSC)

Provide a link to an anonymized genome browser session for "Initial submission" and "Revised version" documents only, to enable peer review. Write "no longer applicable" for "Final submission" documents.

Methodology

Replicates

Describe the experimental replicates, specifying number, type and replicate agreement.

Sequencing depth

Describe the sequencing depth for each experiment, providing the total number of reads, uniquely mapped reads, length of reads and whether they were paired- or single-end.

Antibodies

Describe the antibodies used for the ChIP-seq experiments; as applicable, provide supplier name, catalog number, clone name, and lot number.

Peak calling parameters

Specify the command line program and parameters used for read mapping and peak calling, including the ChIP, control and index files used.

Data quality

Describe the methods used to ensure data quality in full detail, including how many peaks are at FDR 5% and above 5-fold enrichment.

Software

Describe the software used to collect and analyze the ChIP-seq data. For custom code that has been deposited into a community repository, provide accession details.

Flow Cytometry

Plots

Confirm that:

☒ The axis labels state the marker and fluorochrome used (e.g. CD4-FITC).

☒ The axis scales are clearly visible. Include numbers along axes only for bottom left plot of group (a 'group' is an analysis of identical markers).

☒ All plots are contour plots with outliers or pseudocolor plots.

☒ A numerical value for number of cells or percentage (with statistics) is provided.

Methodology

Sample preparation

When indicated, spleen, liver, lung and tumor were harvested from mice and incubated with 0.1 mg collagenase/DNase (Roche) for 30 minutes at 37°C. Single-cell suspensions were prepared by mechanically disrupting the organs through a 70-µm cell strainer (Falcon). Tumor-infiltrating mononuclear cells, liver cells and lung cells were isolated by centrifugation with 44% Percoll (GE Healthcare). Cells were then subjected to red blood cell lysis (Sigma-Aldrich). Blood was collected in PBS containing 2% FCS, 0.1% sodium azide and 2.5 U/ml heparin. Peripheral blood mononuclear cells (PBMCs) were prepared by lysing erythrocytes with red blood cell lysis buffer. Mononuclear cells from the abovementioned organs were washed, resuspended in RPMI+2% FCS (Gibco), counted and kept on ice until further analysis.

Instrument

BD LSR-II

Software

BD FACS Diva

Cell population abundance

No sorting was involved in this study

Gating strategy

Blood: FSC-H/SSC-H (leukocytes)--> FSC-H/FSC-A (Single cells)--> CD4/CD8 (Leukocytes: CD8+)--> OVA Dex/CD44 (Ova-specific CD8 T cells).
Tumor and spleen leukocytes: FSC-H/SSC-H (leukocytes)--> FSC-H/FSC-A (Single cells)--> Live/dead /FSC-H (Live/Dead negative= alive cells)--> SSC-A/ CD45 (CD45+ = tumor infiltrating leukocytes).
Blood, liver, lung and spleen NK cells: FSC-H/SSC-H (leukocytes)--> FSC-H/FSC-A (Single cells)--> Live/dead /FSC-H (Live/Dead negative= alive cells)--> NKp46/CD3 (NK cells= CD3- NKp46+).
Blood T cell and NK cells: FSC-H/SSC-H (leukocytes)--> FSC-H/FSC-A (Single cells)--> CD3/NKp46 (NK cells= CD3- NKp46+). On CD3 + -->CD4/CD8 (Leukocytes: CD8+)--> OVA Dex/CD44 (Ova-specific CD8 T cells). On CD44+ OVA Dex negative--> B8R Dex/CD44 (Vector-specific CD8 T cells).
Blood B cells-->FSC-H/SSC-H (leukocytes)--> FSC-H/FSC-A (Single cells)--> CD3/CD19 (B cells= CD3- CD19+). On CD3- CD19+ --> MHC-II/CD45R (B cells= MHC-II+ CD45R+).

☒ Tick this box to confirm that a figure exemplifying the gating strategy is provided in the Supplementary Information.

Magnetic resonance imaging

Experimental design

Design type

Indicate task or resting state; event-related or block design.

Design specifications	*Specify the number of blocks, trials or experimental units per session and/or subject, and specify the length of each trial or block (if trials are blocked) and interval between trials.*
Behavioral performance measures	*State number and/or type of variables recorded (e.g. correct button press, response time) and what statistics were used to establish that the subjects were performing the task as expected (e.g. mean, range, and/or standard deviation across subjects).*

Acquisition

Imaging type(s)	*Specify: functional, structural, diffusion, perfusion.*
Field strength	*Specify in Tesla*
Sequence & imaging parameters	*Specify the pulse sequence type (gradient echo, spin echo, etc.), imaging type (EPI, spiral, etc.), field of view, matrix size, slice thickness, orientation and TE/TR/flip angle.*
Area of acquisition	*State whether a whole brain scan was used OR define the area of acquisition, describing how the region was determined.*

Diffusion MRI ☐ Used ☒ Not used

Preprocessing

Preprocessing software	*Provide detail on software version and revision number and on specific parameters (model/functions, brain extraction, segmentation, smoothing kernel size, etc.).*
Normalization	*If data were normalized/standardized, describe the approach(es): specify linear or non-linear and define image types used for transformation OR indicate that data were not normalized and explain rationale for lack of normalization.*
Normalization template	*Describe the template used for normalization/transformation, specifying subject space or group standardized space (e.g. original Talairach, MNI305, ICBM152) OR indicate that the data were not normalized.*
Noise and artifact removal	*Describe your procedure(s) for artifact and structured noise removal, specifying motion parameters, tissue signals and physiological signals (heart rate, respiration).*
Volume censoring	*Define your software and/or method and criteria for volume censoring, and state the extent of such censoring.*

Statistical modeling & inference

Model type and settings	*Specify type (mass univariate, multivariate, RSA, predictive, etc.) and describe essential details of the model at the first and second levels (e.g. fixed, random or mixed effects; drift or auto-correlation).*
Effect(s) tested	*Define precise effect in terms of the task or stimulus conditions instead of psychological concepts and indicate whether ANOVA or factorial designs were used.*

Specify type of analysis: ☐ Whole brain ☐ ROI-based ☐ Both

Statistic type for inference<br>(See Eklund et al. 2016)	*Specify voxel-wise or cluster-wise and report all relevant parameters for cluster-wise methods.*
Correction	*Describe the type of correction and how it is obtained for multiple comparisons (e.g. FWE, FDR, permutation or Monte Carlo).*

Models & analysis

n/a	Involved in the study
☒	☐ Functional and/or effective connectivity
☒	☐ Graph analysis
☒	☐ Multivariate modeling or predictive analysis
